# On a Successful Method of Treating Acute Rheumatism by Large and Frequent Doses of the Bicarbonate of Potash

**Published:** 1855-04

**Authors:** A. B. Garrod

**Affiliations:** Physician to University College Hospital


*On a Successful Method of treating Acute Rheumatism by large and frequent doses of the Bicarbonate of Potash.* By A. B. Garrod, M.D., Physician to University College Hospital.—The author, after a few preliminary remarks, observed, that he was induced, in May, 1852, to try a new method of treating acute rheumatism; and finding great success at first, resolved steadily to pursue the plan, and has done so up to the present time. The object of his communication has been to record the method adopted by him, and also the results obtained in fifty-one cases of rheumatic fever which have been admitted, under his care, in University College Hospital, during the last two years and three quarters, The main part of his plan of treatment consists in the administration. in a diluted form, of two-scruple doses of bicarbonate of potash, every two hours, day and night, until the patient has been free from all articular affection and febrile disturbance for two or three days, using local depletion over the heart's region, if any cardiac disease is present or threatened. The author then detailed three cases of rheumatic fever, illustrating this mode of treatment: the first, a girl, ten years old, in which the duration under treatment was five days, the total duration eight; the second, a young man, aged twenty, with a complication of heart disease, where the duration under treatment was eight, the total duration fifteen days; the third, a young woman, aged eighteen years, in the fifth attack, the former ones having always lasted for a month or five weeks, but which, by the adoption of this plan, yielded in nine days, total duration being but thirteen days, four having elapsed before her admission into the hospital. He afterwards gave a table of fifty-one cases of acute rheumatism; and of each patient the following particulars are noted:—The age, occupation, hereditary predisposition; the number and causes of attack; the symptoms before admission; the symptoms during treatment; the nature of treatment; and the duration of the disease. From these cases, the following deductions are made—viz., that in twenty males the duration of the disease under treatment averaged between six and seven days, and the total duration between eleven and twelve days; and in thirty-one females, the disease under treatment averaged from seven to eight days, and the total duration between fifteen and sixteen days—giving in all an average under treatment of seven days and a half; and, for the total duration, about thirteen days and a half. The author then alluded to the influence of the bicarbonate of potash when administered in large and frequent doses upon the different organs and functions of the body; and remarked, that it produces neither nausea, vomiting, nor purging—in fact, no symptom of gastro-intestinal irritation. It now induces a strongly alkaline condition of the urine, causes it to effervesce freely, with excess of acid, but does not appear to promote an increase in the quantity of the secretion. It appears to render the secretion of the skin less acid—sometimes almost neutral. That it acts as a powerful controller of the heart's action, reducing greatly the frequency of the pulse, but without causing the faintness often produced by digitalis, colchicum, &c. That it probably increases the alkalinity of the serum of the blood, and diminishes the coagulability of the altered fibrine occurring in rheumatic fever; and hence, probably, checking or preventing the deposits of lymph on the endo- or peri-cardium. He (Dr. Garrod) stated his opinion, that the influence of the bicarbonate was felt not only in shortening the duration of the articular affection, but also in preventing or moderating the cardiac disease. After enumerating many details of the method adopted, and the value of certain adjuncts, as opium, calomel, and occasional general depletion, he proceeded to recommend a plan of treatment which, from his experience, he considered calculated to ensure the greatest amount of success, and thought it probable that the total duration of the disease might, on the average, be reduced to about ten days,—provided that the treatment was adopted early, and no serious complication existed.—*London Lancet.*

